# Experimental Infection of Foot and Mouth Disease in Indian Sheep and Goats

**DOI:** 10.3389/fvets.2020.00356

**Published:** 2020-06-25

**Authors:** Madhanmohan Muthukrishnan, Nagendrakumar Singanallur Balasubramanian, Srinivasan Villuppanoor Alwar

**Affiliations:** Foot and Mouth Disease Virus Laboratory, Research and Development Centre, Indian Immunologicals Limited, Hyderabad, India

**Keywords:** foot-and-mouth disease, serotype O, experimental infection, sheep, goats

## Abstract

Foot-and-mouth disease (FMD) is an economically important contagious disease of livestock mainly cattle, buffalo, sheep, goats, and pig. There is limited data available on pathogenesis of foot and mouth disease in goats. In the study, the sheep and goats were infected experimentally with a serotype O foot-and-mouth disease virus by different challenge routes. The sheep and goats challenged by coronary band route and coronary band and intra-dermo-lingual route exhibited FMD clinical signs at 2–5 days post challenge. Whereas intra-dermo-lingual challenged sheep and goats did not exhibit FMD clinical signs. Live virus could be isolated from blood of infected sheep and goats at 2–5 days post challenge. Viral RNA could be detected from blood of infected sheep and goats at 1–10 days post challenge. The neutralizing antibody titre was detected at 10 days post challenge and maintained up to 35 days post challenge in all infected sheep and goats. Non structural protein (NSP) antibodies were detected as early as 5–10 days post challenge and remain positive up to 35 days post challenge in the infected sheep and goats. In conclusion, the pathogenesis of sheep and goats with serotype O foot and mouth disease virus by different challenge routes could be demonstrated.

## Introduction

Foot-and-Mouth Disease (FMD) is an infectious disease which causes severe economic loss to the livestock sector (1). FMD is caused by FMD virus (FMDV), a member of family *Picornaviridae* and genus *Aphthovirus* affecting all the cloven footed animals. FMDV exists as seven distinct serotypes viz., O, A, C, Asia 1, Southern African territory 1(SAT1), SAT2, and SAT3. In India, incidence of FMD is reported throughout the country with the prevalence of FMDV serotypes O, A and Asia 1 (2). Cattle and buffalo are vaccinated biannually with inactivated FMD trivalent vaccine to control FMD in India. However, sheep and goats are not included in FMD control program (3, 4). Sheep and goats play an important role in the livelihood of a large percentage of small and marginal farmers and landless laborers in India. India constitutes around 148.88 million heads of goat population and 74.26 million heads of sheep population in the world. Moreover, cattle, buffalo, sheep and goats are grazed together in India (5). FMD outbreaks in sheep and goats are reported in India (6, 7).

There is paucity of information on the role of goats in FMD epidemiology and transmission. FMD infected sheep and goats transmitted the sub-clinical infection to cattle, buffalo, sheep and goats and FMD vaccination in sheep and goats could prevent the transmission of FMD to cattle, buffalo, sheep and goats (8). Usually, sheep and goats showed mild or unapparent FMD clinical signs (9, 10). Moreover, FMD infected goats showed typical oral and foot lesions in India (11). However, there is no detailed account of the pathogenesis of the disease in these small ruminants, especially in goats. This preliminary report describes pathogenesis of sheep and goats experimentally infected with type O foot and mouth disease virus using different challenge routes.

## Materials and Methods

### Cell Line and Viruses

Baby Hamster kidney (BHK) and primary bovine thyroid (BTY) cells were provided by the tissue culture laboratory at Research and Development Centre, Indian Immunologicals Limited (IIL), Hyderabad. BTY cells were grown using Hely cell growth medium supplemented with 10% adult bovine serum and antibiotics cocktail (penicillin, neomycin and polymyxin). O/IND/R2/75 virus was received from the virus seed laboratory, IIL, Hyderabad.

### Experimental Animals

Eight Nellore sheep and eight Osmanabadi goats of either sex (6–12 months of age) were obtained from the holding farm of IIL, Hyderabad. These animals were reared in the farm from one month of age and were screened by three rounds of testing for FMDV-non-structural protein (NSP) antibodies using PrioCHECK® FMDV NS kit (Prionics Lelystad B.V., The Netherlands). All the animals were NSP seronegative in all the three tests. Additionally, the animals were tested for the absence of virus in the oesophagopharyngeal fluids (Probang samples) thrice by virus isolation on primary bovine thyroid cells (12) followed by antigen ELISA (13) and RT-PCR (14).

### Challenge Virus Preparation

Challenge virus O/IND/R2/75 was prepared and titrated by standard methods as described previously (15).

### Experimental Design

One sheep and goat each were inoculated with O/IND/R2/75 cattle challenge virus by intra-dermo-lingual, coronary band and by both sites in 0.1 ml quantity in each site. The animals were monitored for 24–72 h for signs of FMD (passage 1). For a second passage, epithelial tissue collected from vesicles was triturated in 0.04 M phosphate buffer followed by centrifugation at 3000 xg. The clear supernatant was used to inoculate one sheep and goat each by intra-dermo-lingual, coronary band and by both sites in 0.1 ml quantity in each site respectively. The animals were monitored for 24–72 h for signs of FMD. Two sheep and two goats was included as unchallenged control and maintained throughout the study period. Experiments were conducted in a bio-secure animal isolation unit at IIL, Hyderabad. The studies involving animals were reviewed and approved by Institutional Animal Ethics Committee, Indian Immunologicals Limited, Hyderabad and Committee for the Purpose of Control and Supervision of Experiments on Animals (CPCSEA), Department of Animal Husbandry and Dairying, Government of India.

### Clinical Scoring

The sheep and goats were observed for clinical signs of disease and temperatures recorded daily. A subjective scoring system (16) was used to evaluate the progression of disease in these animals with slight modification (8).

### Sample Collection and Processing

Clotted blood for serology and NSP antibody was collected at days 0, 5, 10, 15, 21, 28, and 35 post-challenge. Heparinized blood was collected daily up to 10 dpc. Heparinized blood (200 μl) was mixed with 300 μl of lysis buffer (Roche Diagnostics, Germany) for analysis by real-time RT-PCR (qRT-PCR) and stored at −70°C. Heparinized blood (1 ml) was used for virus isolation (VI) (15).

### Virus Isolation

Heparinized blood samples were examined for the presence of live virus by primary bovine thyroid (BTY) cell culture inoculation (9). BTY tubes were inoculated with 250 μl sample (5 tubes per sample) and incubated in a stationary position for 30 min at 37°C. The tubes were then gently washed with 0.04 M phosphate buffer containing antibiotics and 2 ml of virus maintenance medium was added prior to incubation at 37°C on roller drums. At 24, 48, and 72 h post inoculation, cell monolayer was examined for cytopathic effect (CPE). The presence of FMDV in cultures showing CPE was confirmed using an antigen ELISA (10). BTY cell culture supernatants from samples showing no sign of CPE after 72 h were pooled and re-passaged once and the absence of FMDV was confirmed by the antigen ELISA as mentioned above (15).

### Virus Neutralizing Antibody Test (VNT)

Virus neutralization tests were performed for the sera in flatbottomed tissue culture grade micro titre plates (Nunclon™, Denmark) as described previously (17). Antibody titres were expressed as the reciprocal of the final dilution of serum in the serum/virus mixture which neutralized an estimated 100 TCID_50_ of virus at the 50% end-point (18).

### Non Structural Protein Antibody Test

Antibodies to FMDV NSP 3ABC were tested using PrioCHECK®FMDV NS kit (Prionics Lelystad B.V., The Netherlands) (19).

### Quantitative Real-Time RT-PCR Assay for Detection of Viral RNA

The amount of viral RNA in blood was quantified by qRT-PCR (20). The total nucleic acid was extracted from liquid samples with MagNApure LC total nucleic acid isolation kit (Roche Diagnostics GmbH, Germany) using an automated nucleic acid robotic workstation (MagNApure LC, Roche Diagnostics GmbH, Germany). For the generation of standard curves, a FMDV RNA standard was synthesized *in vitro* from a plasmid containing a 79 base pair insert of the internal ribosomal entry site (IRES) of a type O FMDV (kindly provided by Dr. Donald P. King, Institute for Animal Health, UK) using a MEGAscript® T7 kit (Ambion, USA) as described previously (21) in an IQ®5 Multicolor Real-time PCR detection system (BioRad, USA). The results from all samples were analyzed using Bio-Rad iQ®5 optical system software and CT values were assigned to each reaction (18). Viral RNA was quantified using a standard curve derived from the standard RNA preparation at different concentrations (10^8^-10^1^) (22).

## Results

### Development of Clinical FMD

**Figure 1:**
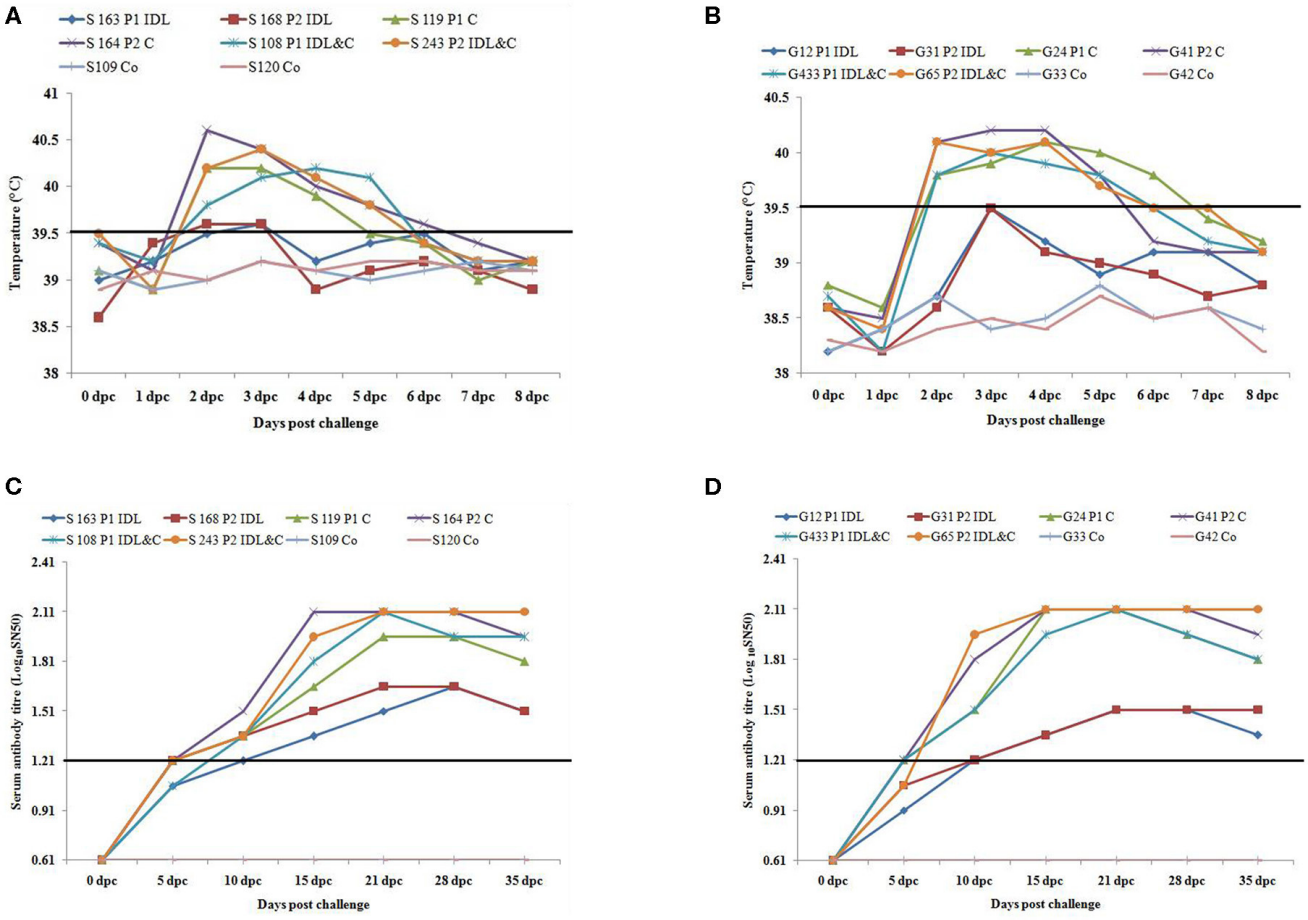
**(A)** Rectal temperature (°C) of challenged (intra-dermo-lingual (IDL), coronary band (C) and by both route) and control sheep. P1, passage 1; P2, Passage 2. Solid line indicates normal temperature of sheep. **(B)**. Rectal temperature (°C) of challenged (intra-dermo-lingual (IDL), coronary band (C) and by both route) and control goats. P1, passage 1; P2, Passage 2. Solid line indicates normal temperature of goats. **(C)** Virus neutralization titres of challenged and control sheep (Expressed as the log10 reciprocal antibody dilution required for 50% neutralization of 100 tissue culture infectious units). Solid line indicates neutralizing antibody titre >1.2 log_10_SN50 is considered positive. **(D)** Virus neutralization titres of challenged and control goats (Expressed as the log10 reciprocal antibody dilution required for 50% neutralization of 100 tissue culture infectious units). Solid line indicates neutralizing antibody titre >1.2 log_10_SN50 is considered positive.

Sheep inoculated by intra-dermo-lingual route did not exhibit clinical signs of FMD in both passages. Sheep inoculated by coronary band route showed clinical signs of FMD such as inappetance, panting, pyrexia (≥40 °C) (Figure 1A), lameness and vesicles in foot and mouth at 2–5 dpc. Sheep inoculated by both intra-dermo-lingual/coronary band routes showed FMD clinical signs at 5 dpc. Goats inoculated by intra-dermo-lingual route did not exhibit clinical signs of FMD in both passages. Goats inoculated by coronary band route showed clinical signs of FMD such as inappetance, panting, pyrexia (≥40 °C) (Figure 1B), lameness and vesicles in foot and mouth at 2–5 dpc. Goats inoculated by both intra-dermo-lingual/coronary band routes also showed FMD clinical signs at 3 dpc. The unchallenged control sheep and goats did not show any FMD clinical signs (Table 1).

**Table 1:** Lesion score of challenged and control sheep and goats.

**Groups**	**Animal No**	**0 dpc**	**1 dpc**	**2 dpc**	**3 dpc**	**4 dpc**	**5 dpc**	**6 dpc**	**7 dpc**	**8 dpc**
Intra-dermo-lingual	S163 P1	0	0	0	0	0	0	0	0	0
	S168 P2	0	0	0	0	0	0	0	0	0
	G12 P1	0	0	0	0	0	0	0	0	0
	G31 P2	0	0	0	0	0	0	0	0	0
Coronary band	S119 P1	0	0	1	3	3	3	2	0	0
	S164 P2	0	0	2	5	6	6	5	4	4
	G24 P1	0	0	1	2	2	2	0	0	0
	G41 P2	0	0	1	4	6	6	5	3	0
Intra-dermo-lingual and coronary band	S108 P1	0	0	0	0	0	0	0	0	0
	S243 P2	0	0	1	2	2	3	2	2	0
	G433 P1	0	0	1	1	2	0	0	0	0
	G65 P2	0	0	1	3	2	1	0	0	0
Control	S109	0	0	0	0	0	0	0	0	0
	S120	0	0	0	0	0	0	0	0	0
	G 33	0	0	0	0	0	0	0	0	0
	G42	0	0	0	0	0	0	0	0	0

*S, Sheep; G, Goats; P1, Passage 1; P2, Passage 2; dpc, days post challenge*.

*Lesion in inoculated feet−1; Mouth lesions−2; Lesion in one foot other than the inoculated foot−2; lesion in two feet other than the inoculated feet−3; lesion in three feet other than the inoculated feet−4. The scores were then added*.

### Detection of Virus/ Virus Nucleic Acid in Blood

In passage 1, virus could not be isolated form challenged sheep and goats (S163, S119, S108, G12, G24, and G433) irrespective of challenge routes. In passage 2, infectious virus was isolated form intra-dermal-lingual route challenged sheep (S168) and goat (G31) at 3 dpc and coronary band challenged sheep (S164) and goat (G41) at 2–5 dpc. Whereas in intra-dermal-lingual and coronary band route challenged sheep (S243) was positive for virus isolation at 4–5 dpc. In the case of intra-dermal-lingual and coronary band route challenged goat (G65) virus was isolated on 2 and 5 dpc.

Viral RNA (10^8.44^–10^9.45^ viral RNA copy numbers/ml of blood) was detected as early as 1 dpc from the sheep (S119P1 and S164P2) and goat (G41P2) inoculated by coronary route. Viral RNA (10 ^9.42^-10 ^10.47^) was detected in all the inoculated sheep between 1 and 10 dpc irrespective of challenge routes. Viral RNA (10 ^7.42^-10 ^9.82^) was detected in all the inoculated goats between 1 and 10 dpc. All the unchallenged control sheep and goats were negative for virus isolation and viral RNA from blood samples (Table 2).

**Table 2:** Virus isolation and quantification of FMD viral RNA copy numbers (Log_10_ RNA copy numbers/ ml of blood) from blood of challenged and control sheep and goats.

**Groups**	**Sheep/ goats**	**0 dpc**		**1 dpc**		**2 dpc**		**3 dpc**		**4 dpc**		**5 dpc**		**6 dpc**		**7 dpc**		**8 dpc**		**9 dpc**		**10 dpc**	
		**VI**	**PCR**	**VI**	**PCR**	**VI**	**PCR**	**VI**	**PCR**	**VI**	**PCR**	**VI**	**PCR**	**VI**	**PCR**	**VI**	**PCR**	**VI**	**PCR**	**VI**	**PCR**	**VI**	**PCR**
Intra-dermo-lingual	S 163 P1	-	0	-	0	-	9.46	-	9.47	-	9.43	-	0	-	0	-	9.4	-	0	-	0	-	0
	S 168 P2	-	0	-	0	-	9.45	+	9.42	-	10.5	-	0	-	0	-	0	-	0	-	0	-	9.43
	G12 P1	-	0	-	0	-	0	-	0	-	9.43	-	9.48	-	0	-	0	-	0	-	0	-	0
	G31 P2	-	0	-	0	-	0	+	9.42	-	0	-	0	-	0	-	0	-	0	-	0	-	0
Coronary band	S 119 P1	-	0	-	9.45	-	9.44	-	0	-	0	-	0	-	0	-	0	-	0	-	0	-	0
	S 164 P2	-	0	-	9.44	+	9.44	+	9.42	+	9.51	+	9.57	-	0	-	9.51	-	0	-	0	-	9.42
	G24 P1	-	0	-	0	-	9.44	-	8.45	-	0	-	9.45	-	0	-	0	-	0	-	0	-	0
	G41 P2	-	0	-	8.44	+	8.66	+	9.82	+	9.61	+	9.37	-	0	-	8.42	-	0	-	0	-	7.42
Intra-dermo-lingual And coronary band	S 108 P1	-	0	-	0	-	9.49	-	0	-	0	-	0	-	0	-	0	-	0	-	0	-	0
	S 243 P2	-	0	-	0	-	9.57	-	9.52	+	9.42	+	9.48	-	0	-	0	-	0	-	0	-	0
	G433 P1	-	0	-	0	-	8.49	-	0	-	0	-	0	-	0	-	0	-	0	-	0	-	0
	G65 P2	-	0	-	0	+	8.57	-	8.52	-	8.42	+	8.48	-	0	-	0	-	0	-	0	-	0
Control	S109	-	0	-	0	-	0	-	0	-	0	-	0	-	0	-	0	-	0	-	0	-	0
	S120	-	0	-	0	-	0	-	0	-	0	-	0	-	0	-	0	-	0	-	0	-	0
	G 33	-	0	-	0	-	0	-	0	-	0	-	0	-	0	-	0	-	0	-	0	-	0
	G42	-	0	-	0	-	0	-	0	-	0	-	0	-	0	-	0	-	0	-	0	-	0

*S, Sheep; G-Goats; P1, Passage 1; P2, Passage; + Positive for virus isolation; Negative for virus isolation; dpc, days post challenge*.

### FMDV NSP Antibody Response

Four inoculated sheep (S163, S119, S108 and S243) were positive for NSP antibody on 10 dpc while other sheep NSP antibodies were observed on 15–35 dpc. Three inoculated goats (G12, G31 and G433) were positive for NSP antibody on 5 dpc while in other goats NSP antibodies were observed on 15–35 dpc. Both the unchallenged control sheep and goats were negative for NSP antibody up to 35 dpc (Table 3).

**Table 3:** FMDV NSP antibody responses of challenged and control sheep and goats.

**Groups**	**Animal No**	**0 dpc**	**5 dpc**	**10 dpc**	**15 dpc**	**21 dpc**	**28 dpc**	**35 dpc**
Intra-dermo-lingual	S163 P1	N	N	P	P	P	P	P
	S168 P2	N	N	N	P	P	P	P
	G12 P1	N	P	P	P	P	P	P
	G31 P2	N	P	P	P	P	P	P
Coronary band	S119 P1	N	N	P	P	P	P	P
	S164 P2	N	N	N	P	P	P	P
	G24 P1	N	N	P	P	P	P	P
	G41 P2	N	N	N	P	P	P	P
Intra-dermo-lingual andcoronary band	S108 P1	N	N	P	P	P	P	P
	S243 P2	N	N	P	P	P	P	P
	G433 P1	N	P	P	P	P	P	P
	G65 P2	N	N	P	P	P	P	P
Control	S109	N	N	N	N	N	N	N
	S120	N	N	N	N	N	N	N
	G 33	N	N	N	N	N	N	N
	G42	N	N	N	N	N	N	N

*S, Sheep; G-Goats; P1, Passage 1; P2, Passage 2; P, Positive for NSP antibody; N, Negative for NSP antibody; dpc, days post challenge*.

### Virus Neutralizing Antibody Response

The neutralizing antibody titer was detected in all inoculated sheep and goats at 10 dpc (> 1.2 log_10_SN_50_). However, the highest neutralizing antibody titer was detected between 10 and 35 dpc (2.1 log_10_SN_50_) in sheep and goats inoculated by coronary and both by coronary and intra-dermo- lingual route. Both the unchallenged control sheep and goats had no serum neutralizing antibody titre up to 35 dpc (Figures 1C,D).

## Discussion

The experiment described the preliminary results on pathogenesis and development of FMD in sheep and goats by inoculating the type O FMD virus in three different challenged routes. The development of clinical signs was observed. The viral RNA levels in blood were quantified.

The incubation period of natural FMDV infection is normally between 3 and 8 days in sheep (23), but can be as short as 24 h following experimental infection (23, 24). In the current study, lesions were evident in three sheep and four goats on the 2nd day of challenge.

Sheep and goats inoculated by coronary band route showed clinical signs of FMD such as inappetance, panting, pyrexia (≥40 °C), lameness and vesicles in foot and mouth at 2–5 dpc. This finding was in accordance with the earlier experiments in sheep (25–27) and goats (28). Hughes et al. (29) reported intra nasal inoculation of FMDV also resulted in generalized infection in sheep. Sheep and goats inoculated by both intra-dermo-lingual/coronary band routes also showed FMD clinical signs whereas sheep and goats inoculated by intra-dermo-lingual route did not produce clinical signs of FMD. It should be noted that although sheep and goats inoculated by the intra-dermo-lingual route showed no signs of generalized infection, virus could be isolated from blood samples and viral RNA was detected in blood samples up to 10 dpc. Furthermore, Lazarus, et al. (30) presented that indigenous South African goats manifested FMD clinical signs by challenging intra-dermo-lingual route with SAT1 virus pool. However, the clinical signs of FMD may be influenced by the virus strain and the breed of sheep and goats (31). In the present study, type O virus and Indian breed of sheep and goats were used. This may be the reason for intra-dermo-lingual challenged sheep and goats did not show the clinical signs of FMD.

Intra-dermo-lingual route of inoculation in cattle, dental pad/gum route of inoculation in buffalo (32) and intra dermal inoculation in the heel bulb in pigs (33) of FMDV resulted in generalized disease.

Ryan et al. (27) reported that all inoculated ewes developed viraemia at 1 dpi and viral RNA levels then peaked at 2 dpi. In the current study, viral RNA was detected as early as 1dpc, viral RNA level then peaked at 2–5 dpc from the inoculated sheep and goats. Virus was isolated from blood of inoculated sheep and goats up to 2–5 dpc as reported by Parida et al. (34).

Infection with live foot and mouth disease virus induces non structural antibody response in animals (35). Earlier studies (15, 34) revealed antibodies against 3ABC in sheep at 10 dpc. In the current experiment, all the sheep were positive for NSP antibodies on 10 dpc and continued up to the end of the experiment (35 dpc). In case of goats NSP antibodies were detected as early as 5 dpc and continued up to the end of the experiment (35 dpc). This finding was in accordance with the earlier experiment in sheep and goats (8, 36).

In the present study, neutralizing antibody titre was detected in all the inoculated sheep and goats at 10 dpc and the peak antibody titre was detected between 10 and 35 dpc. Dellers et al. (25) reported neutralizing antibodies were first detected 60 h post inoculation and initial peak titers occurred by 10th day in inoculated sheep.

In this study statistical analysis could not be carried out due to the small number of animals in each group (*n* = 2). In coronary band and Intra-dermo-lingual challenge group of animals received double the dose of challenge virus (0.2 ml) against the Intra-dermo-lingual challenge group (0.1 ml) and coronary band challenge group (0.1 ml), respectively. These are the limitations of this study. So, further study with increased number of animals and statistical analysis is warranted to confirm this result.

## Conclusion

The sheep and goats were infected experimentally with a serotype O foot-and-mouth disease virus by different challenge routes. The sheep and goats challenged by coronary band route and coronary band and intra-dermo-lingual route exhibited FMD clinical signs at 2–5 days post challenge. Whereas intra-dermo-lingual challenged sheep and goats did not exhibit FMD clinical signs. Live virus could be isolated from blood of infected sheep and goats at 2–5 days post challenge. Viral RNA could be detected from blood of infected sheep and goats at 1–10 days post challenge. The neutralizing antibody titre was detected at 10 days post challenge and maintained up to 35 days post challenge in all infected sheep and goats. NSP antibodies were detected as early as 5–10 days post challenge and remain positive up to 35 days post challenge in the infected sheep and goats. In conclusion, the pathogenesis of sheep and goats with serotype O foot and mouth disease virus by different challenge routes could be demonstrated.

## Data Availability Statement

All datasets presented in this study are included in the article/supplementary material.

## Ethics Statement

The studies involving animals were reviewed and approved by Institutional Animal Ethics Committee, Indian Immunologicals Limited, Hyderabad and Committee for the Purpose of Control and Supervision of Experiments on Animals (CPCSEA), Department of Animal Husbandry and Dairying, Government of India.

## Author Contributions

SV designed, coordinated, reviewed and corrected the manuscript. MM and NS performed the experiments, completed analysis and wrote the manuscript. All authors contributed to the article and approved the submitted version.

## Conflict of Interest

MM, NS, and SV were employed by the company Indian Immunologicals Limited, Hyderabad, India.
